# Two new structural mutations in the 5′ region of the *ASIP* gene cause diluted feather color phenotypes in Japanese quail

**DOI:** 10.1186/s12711-019-0458-6

**Published:** 2019-04-15

**Authors:** Annie Robic, Mireille Morisson, Sophie Leroux, David Gourichon, Alain Vignal, Noémie Thebault, Valérie Fillon, Francis Minvielle, Bertrand Bed’Hom, Tatiana Zerjal, Frédérique Pitel

**Affiliations:** 1 GenPhySE, Université de Toulouse, INRA, ENVT, 31326 Castanet-Tolosan, France; 2 INRA PEAT, 37380 Tours, France; 3 GABI, INRA, AgroParisTech, Université Paris-Saclay, 78350 Jouy-en-Josas, France

## Abstract

**Background:**

In quail, two feather colour phenotypes i.e. fawn-2/beige and yellow are associated with the *ASIP* locus. The aim of our study was to characterize the structural modifications within this locus that explain the *yellow* mutation (large deletion) and the *fawn*-2/*beige* mutation (assumed to be caused by a different structural modification).

**Results:**

For the yellow phenotype, we identified a complex mutation that involves a 141,162-bp long deletion. For the fawn-2/beige phenotype, we identified a 71-kb tandem duplication that comprises one unchanged copy of *ASIP* and one copy present in the *ITCH*-*ASIP* fusion gene, which leads to a transcript coding for a normal ASIP protein. Although this agrees with previous reports that reported an increased level of ASIP transcripts in the skin of mutant animals, we show that in the skin from fawn-2/beige embryos, this level is higher than expected with a simple duplication of the *ASIP* gene. Thus, we hypothesize that the 5′ region of the *ITCH*-*ASIP* fusion gene leads to a higher transcription level than the 5′ region of the *ASIP* gene.

**Conclusions:**

We were able to conclude that the fawn-2 and beige phenotypes are caused by the same allele at the *ASIP* locus. Both of the associated mutations *fawn*-*2/beige* and *yellow* lead to the formation of a fusion gene, which encodes a transcript for the ASIP protein. In both cases, transcription of *ASIP* depends on the promoter of a different gene, which includes alternative up-regulating sequences. However, we cannot exclude the possibility that the loss of the 5′ region of the *ASIP* gene itself has additional impacts, especially for the *fawn*-*2/beige* mutation. In addition, in several other species including mammals, the existence of other dominant gain-of-function structural modifications that are localized upstream of the *ASIP* coding sequences has been reported, which supports our hypothesis that repressors in the 5′ region of *ASIP* are absent in the *fawn*-2/*beige* mutant.

**Electronic supplementary material:**

The online version of this article (10.1186/s12711-019-0458-6) contains supplementary material, which is available to authorized users.

## Background

The pigmentation of hair in mammals and of feathers in birds is mainly determined by the relative distribution of two types of melanin, i.e. eumelanin (black/brown) and pheomelanin (yellow/red). The *agouti* (*ASIP*) gene codes for the agouti-signaling protein (ASIP), which is an antagonist of the α-MSH hormone (melanocyte-stimulating hormone) for the melanocortin-1 receptor (MC1R) and leads to decreased eumelanin synthesis in favor of pheomelanin synthesis in melanocytes [1].

In general, (recessive) loss-of-function mutations in the *ASIP* gene, which either impair protein function or reduce its transcription, lead to increased production of eumelanin and to darker coat color as observed in mice [2], rabbits [3] or quails [4]. Conversely, (dominant) gain-of-function mutations that display ubiquitous and constitutive expression of *ASIP*, such as the *lethal yellow* allele in mice, cause a yellow pigmentation [5]. Several studies have shown that structural modifications in the *ASIP* gene are responsible for lighter coat colors in various species: (1) a large deletion in the *lethal yellow* allele in mice [5–7], (2) an insertion in the *A*^*br*^ allele in Normande cattle [8] and in the *viable yellow* allele in mice [9, 10], (3) a tandem duplication in the dominant *white*|*tan* allele in sheep [11], (4) an inverted duplication in the *ASIP* gene in mice [12], and (5) an insertion or an inverted duplication in the *white* allele in Alpaca [13].

An allelic series of variants at the autosomal *yellow* (*Y*) locus, which involves the *ASIP* gene on quail chromosome 20, has been identified in Japanese quail (*Coturnix japonica*): recessive *black* and *yellow* alleles, three alleles (*fawn*, *fawn*-2 and *beige*) that result in three similar phenotypes and the *wild*-*type* allele (*WT*) [14]. The aim of our work was to characterize precisely the molecular basis of two of these variants that cause light plumage colours: *yellow* [15] and *fawn*-2/*beige* [16, 17].

The *yellow* allele is dominant over the *wild*-*type* allele, and homozygous carriers for the *yellow* allele are lethal. Heterozygotes have wheat-straw yellow-coloured feathers [15]. In the literature, the genetic basis of the yellow phenotype is described as a large genomic deletion that spans almost the entire coding sequence of the *RALY* and *EIF2S2* genes upstream of *ASIP* and that probably causes the lethality observed in homozygous individuals. Thus, this deletion places the expression of *ASIP* under the control of the promoter of *RALY* and leads to the expression of a fusion transcript [15]. As a result, ASIP mRNA expression is upregulated in many tissues in heterozygous yellow animals compared to wild-type individuals, although no clearly significant differences were reported in skin samples between these animals [4, 15]. However, the alterations in feather colour are hardly visible and represent only a very small portion of the complex yellow phenotype [18].

The *fawn*-2/*beige* allele is part of a group of alleles responsible for very similar phenotypes that were independently described: *fawn*, *fawn*-2 and *beige*. In Japanese quail, Nichols et al. [19] reported in 1988 a first fawn dilution phenotype with a light/buff coloration and some darker spots. This *fawn* allele was identified as incompletely dominant to *wild*-*type* and co-dominant to *yellow* [20]. Later in 1996, Tsudzuki et al. [17] described a similar fawn-2 phenotype in a population of Japanese quail that had been established at Gifu University from French commercial eggs. In 2003, a beige phenotype similar to fawn-2 was found in a French commercial line of Japanese quail [16]. Both *fawn*-*2* and *beige* alleles are dominant to *wild*-*type* [16, 17] and correspond probably to the same allele. At birth, individuals are characterized by a light yellow plumage with three dark bands on the back (but not on the head) [17]. Adults have a lighter coat color and display a more pronounced sexual dimorphism than wild-type individuals [16, 17] and (see Additional file 1: Figure S1).

Although these studies have contributed to the understanding of the functional activity of *ASIP* in the yellow and the fawn-2/beige phenotypes, a fine genomic characterization of the causative variants is still lacking. The aims of this study were to characterize the large deletion that causes the yellow phenotype and to describe the *fawn*-2/*beige* mutation, which we hypothesized to be due to a structural modification representing a single allele. Thus, we sequenced four individuals i.e. one homozygous beige, one homozygous fawn-2 and two yellow, and compared the data with the recently available C*oturnix japonica* 2.0 quail annotated reference genome. Then, the molecular consequences of the *fawn*-*2/beige* mutation were evaluated.

## Methods

### Whole-genome paired-end sequencing

All animals were produced and maintained at the Inra PEAT experimental unit (Pôle d’Expérimentation Avicole de Tours, authorization # D37–175–1, 2017) in Nouzilly (France) in compliance with the European Union Guidelines for animal care, and with an approval by the local ethical committee in animal experimentation (Val de Loire) and the French Ministry of Higher Education and Scientific Research (authorization # 02411.02). For sequencing, we sampled two heterozygous yellow quails from a line established at Gifu University (Japan) [15] and maintained in Nouzilly, and one homozygous beige and one homozygous fawn-2 individual. DNA was extracted from blood and fragmented in order to build libraries with fragments of ~ 350 bp. Paired-end sequencing (2 × 150 bp) was done following the Illumina TruSeq DNA PCR-free protocol and using an Illumina Hiseq 3000 instrument.

### Alignment of the sequenced fragments to the reference genome

Sequence reads were aligned with BWA-MEM [21] to the *Coturnix japonica* 2.0 quail reference genome (NCBI assembly accession GCA_001577835.1; BioProject accession PRJNA314147), which consists of 32 chromosomes or linkage groups and 1095 un-localised scaffolds [22]. We used the Integrative Genome Viewer (IGV) software http://software.broadinstitute.org/software/igv/ to visualize the alignment on the reference genome and to check for compatibility of distance and orientation between read-pairs with the size selection that was set for the construction of the libraries. IGV was also used to evaluate read depth. Close examination of such alignments in IGV can be very informative to characterise deletions or duplications, for details (see Additional file 2).

### Analysis of the transcripts

Twenty-five beige and 25 wild-type plumage quail eggs were incubated during 15 days before harvesting the embryos. DNA was extracted from muscle, and after sex determination [23] five skin samples of each sex were obtained for each phenotype for further RNA analyses.

Quantitative PCR were performed on the ABI 7900HT (Sequence Detection System 7900HT) in one 384-well plate. Since efficiency levels were similar for all the genes measured (including the reference genes *RPS13* and *GAPDH*), results were expressed as 2^(Ct_ref  − Ct_gene)^ × 1000 in arbitrary units. For a complete description (see Additional file 3 and Additional file 4: Table S1).

## Results

### Annotation of the region carrying the *ASIP* gene

**Fig. 1:**
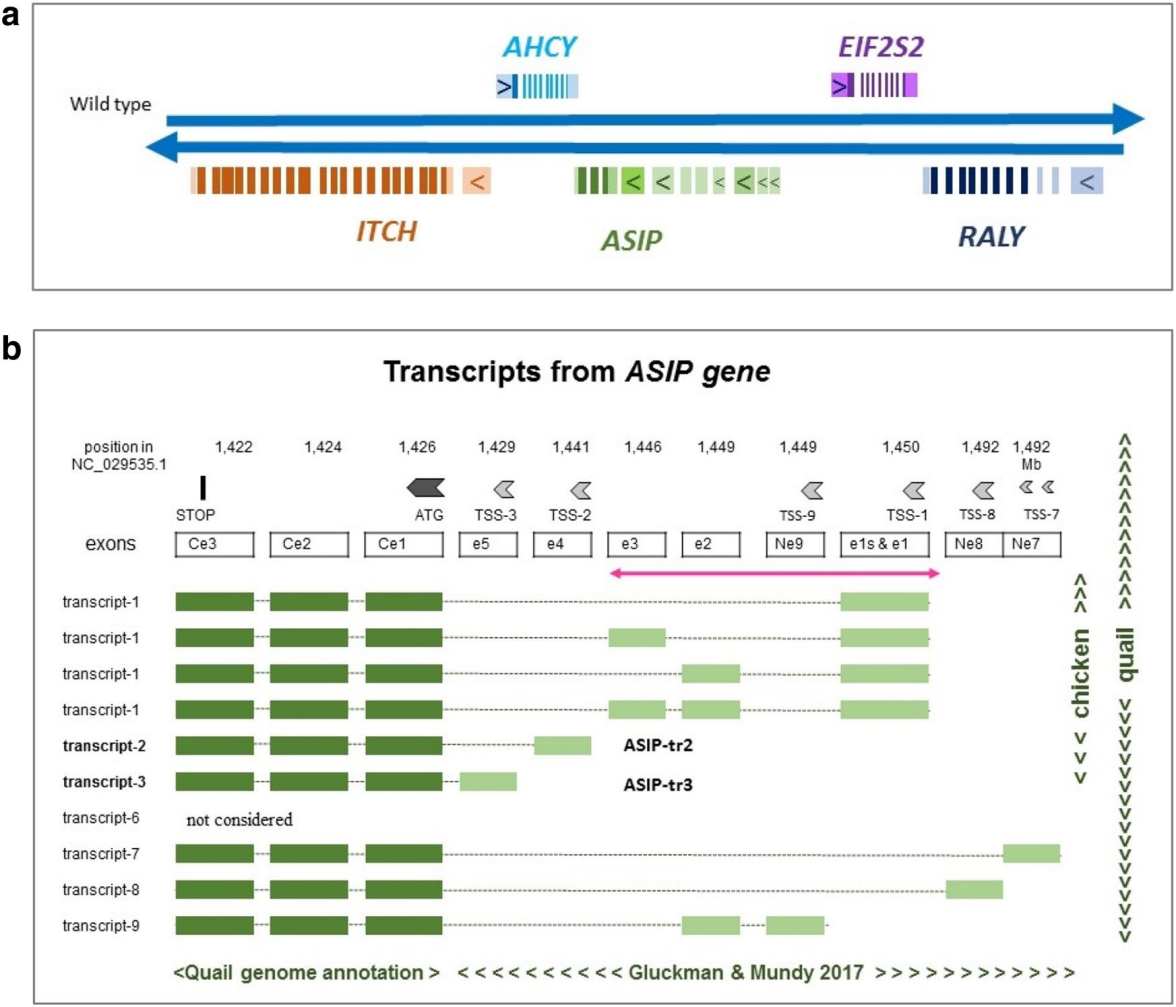
*ASIP* locus in quail. **a** Schematic representation of the *ASIP* locus in quail. Each gene is represented with a specific color: the coding exons are schematized by dark rectangles while the 5′ UTR exons are represented in a lighter shade. The 5′ UTR exons with potential transcription start sites are symbolized by a rectangle with an arrow. **b** Presentation of the *ASIP* gene and ASIP transcripts. We expect nine types of transcripts encoding the same ASIP protein. For the exons, we used the names proposed by Gluckman and Mundy [23]. The coding exons are Ce1, Ce2 and Ce3. We did not consider the ASIP transcript-6 as proposed by [23], because the sequence of the 5′ UTR exon relative to this transcript (novel6) was not included in this region of quail chromosome 20. The double pink arrow shows the 21.4-kb region highlighted in this study (see also Fig. 3)

We used the newly annotated *Coturnix japonica* 2.0 reference genome to improve the characterization of the region that carries the *ASIP* gene. *ASIP* was on the same strand as *ITCH* and *RALY*, while *AHCY* and *EIF2S2* were on the other strand (Fig. 1a). In the reference sequence, *ITCH, RALY* and *AHCY* each harboured only one described transcription start site (TSS) in their unique untranslated exon 1 (5′UTR). The *ASIP* gene was correctly annotated for its three well described coding exons Ce1, Ce2 and Ce3 as previously reported [24]. In chicken, *ASIP* has at least three possible upstream promoters with three TSS, i.e. TSS-1, TSS-2 and TSS-3 (Fig. 1b). Recent studies [15, 23] showed that three other possible TSS (not reported to date in chicken) exist in quail, namely TSS-7, TSS-8 and TSS-9 (Fig. 1b).

### Characterization of the *yellow* mutation

**Fig. 2:**
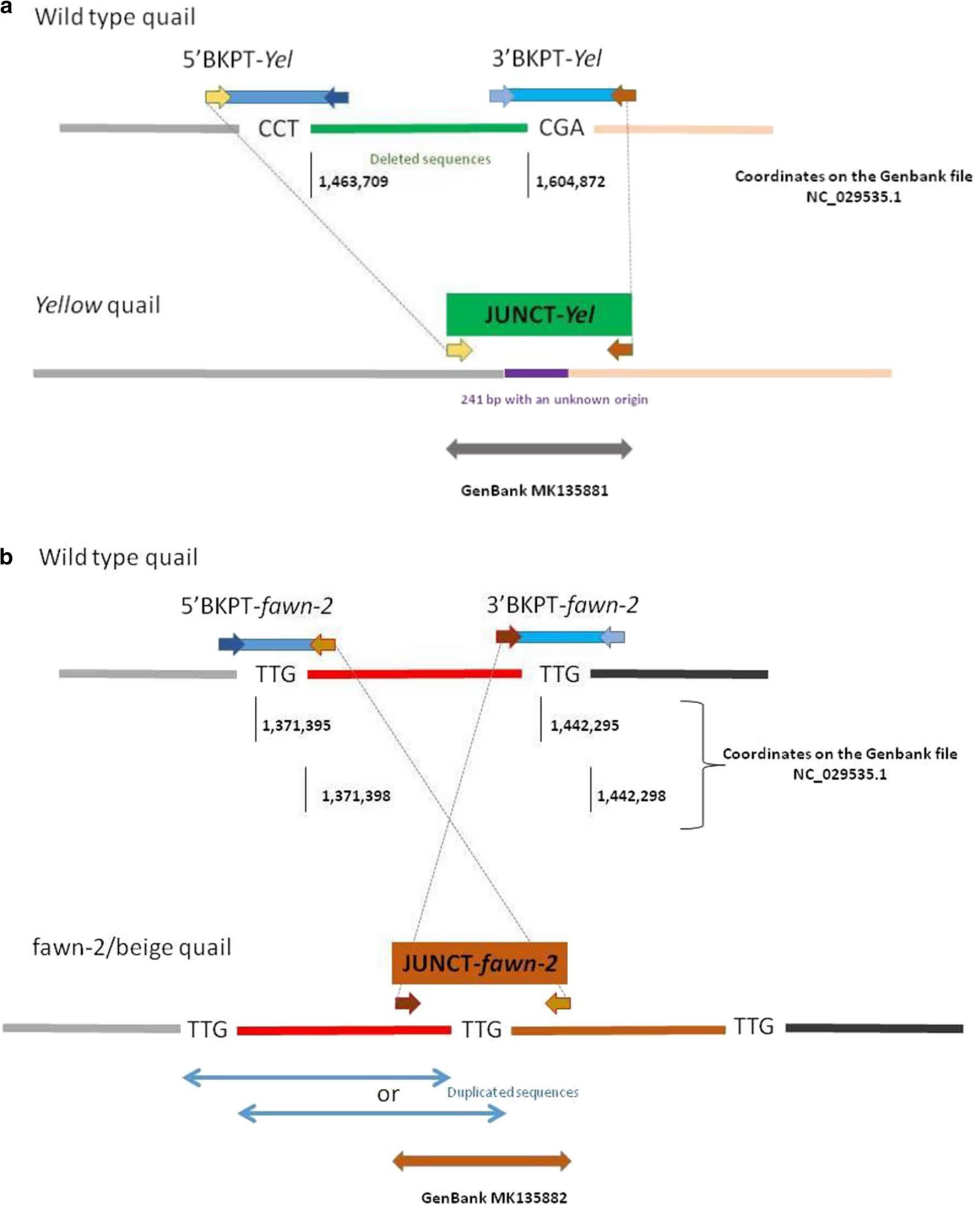
Characterization of the *yellow* and *fawn*-2/*beige* mutations. **a** Schematic characterization of the genomic region in quail carrying the *yellow* deletion. The breakpoint upstream from the deletion was named 5′BKPT-*Yel* (for 5′ breakpoint), the downstream breakpoint was named 3′BKPT-*Yel*, and the junction point was named JUNCT-*Yel*. The colored arrows represent the different primer pairs used to validate the variant with a deletion. The fragment JUNCT-*Yel* was sequenced and includes an insertion of 241 bp of unknown origin (represented in purple). **b** Schematic characterization of the genomic region in quail carrying the *fawn*-2/*beige* tandem duplication. The breakpoints upstream and downstream from the duplication were named 5′BKPT-*fawn*-*2* and 3′BKPT-*fawn*-*2*, respectively, and the junction point was named JUNCT-*fawn*-2. The colored arrows represent the different primer pairs used to validate the variants with a deletion. The fragment JUNCT-*fawn*-2 was sequenced and is represented in brown

Paired-end reads obtained from two heterozygous yellow individuals (SRA biosamples SRR8224502 and SRR8224503) were analysed with IGV within the region including *ASIP* on quail chromosome 20. We found two paired-end reads that potentially detected a large deletion (see Additional file 5: Figure S2) as expected from previous studies [15]. The breakpoint upstream from the putative deletion was named 5′BKPT-*Yel* (for the 5′ breakpoint), the downstream breakpoint 3′BKPT-*Yel*, and the junction point JUNCT-*Yel* (Fig. 2a). Among the reads that map to these two breakpoint regions, several split reads were observed (see Additional file 5: Figure S2). After individual mapping with BLAST [25], these reads did not appear to be segmented around the deletion as expected for a simple deletion (see Additional file 5: Figure S2): the first segment was located upstream or downstream of the deletion but the second segment did not map to this chromosome, which suggested a more complex event. To better understand the rearrangement, the three regions (3′ and 5′ BKPT-*Yel*, and JUNCT-*Yel*) were amplified from two yellow heterozygous individuals. For the 3′ and 5′ BKPT-*Yel*, amplified fragments had the expected size, but for JUNCT-*Yel* the amplified fragment was longer than expected and, thus, was sequenced (GenBank MK135881). BLAST alignment on the quail reference genome revealed that the JUNCT-*Yel* sequence comprised three fragments: the 5′ end mapped upstream to the 5′BKPT-*Yel* and the 3′ end mapped downstream of the 3′BKPT-*Yel*, which was consistent with a 141,162-bp deletion (Fig. 2a) (NC-029535 [1,463,709–1,604,872]), while the third fragment aligned with an unexpected internal fragment of 241 bp (Fig. 2a). BLAST and BWA-MEM were not able to propose a unique position for this 241-bp fragment on the quail reference genome, but showed that it was present within the 141,162-bp deletion. The fact that this 241-bp sequence was present at multiple locations in the genome probably explains why we found only two paired-end reads (2×150 bp) encompassing the 141,162-bp deletion, i.e. most of the reads from this region probably mapped to various sites (see Additional file 5: Figure S2). After sequencing, no microhomology (identity of a short nucleotide sequence in two non-complementary DNA strands) was observed at the borders of the deletion. We used the 3′BKPT-*Yel* and JUNCT-*Yel* fragments to genotype the deletion in 50 wild-type or fawn-2/beige quails and two quails heterozygous for the *yellow* mutation. As expected (Fig. 2a), 3′BKPT-*Yel* (i.e. the positive PCR control) was found in all animals and JUNCT-*Yel* was found only in animals carrying the y*ellow* mutation (Table 1).

**Table 1:** Validation of both structural modifications as the causal mutation

	Wild type	Homozygous		Heterozygous *yellow*
		*fawn*-*2*	*beige*	
3′BKPT-*Yel*	38/38	2/2	10/10	2/2
JUNCT-*Yel*	0/38	0/2	0/10	2/2
3′BKPT-*fawn*-*2*	30/30	2/2	25/25	2/2
JUNCT-*fawn*-*2*	0/30	2/2	25/25	0/2

### Characterization of the *fawn-2/beige* mutation

By examining the alignments of paired-ends obtained from the fawn-2 (SRA biosample SRR8224504) and beige individuals (SRR8224505), we found several paired-ends that had reads in opposite orientations (tail to tail) and that were separated by a large distance from each other in both individuals (see Additional file 5: Figure S3). This suggests a tandem duplication in the *ASIP* region. The increase in read depth for this region is in favour of a simple tandem duplication [the possibility of three or four copies of this region was excluded, (see Additional file 5: Figure S3)]. The breakpoints that were located upstream and downstream of the duplication were named 5′BKPT-*fawn*-*2* and 3′BKPT-*fawn*-*2*, respectively and the junction point was named JUNCT-*fawn*-2 (Fig. 2b). These three regions were amplified from one homozygous fawn-2 and one homozygous beige individual and sequenced, and a 3-bp (TTG) microhomology was detected in each of these three regions (Fig. 2b). We used the 3′BKPT-*fawn*-*2* and JUNCT-*fawn*-2 fragments to validate the presence of the duplication in fawn-2 and beige animals (Table 1). Based on our findings, we can conclude that *fawn*-*2* and *beige* are the same allele, and that it is caused by a 70,895-bp tandem duplication.

### Consequences of the 70,895-bp tandem duplication in fawn-2/beige individuals

**Fig. 3:**
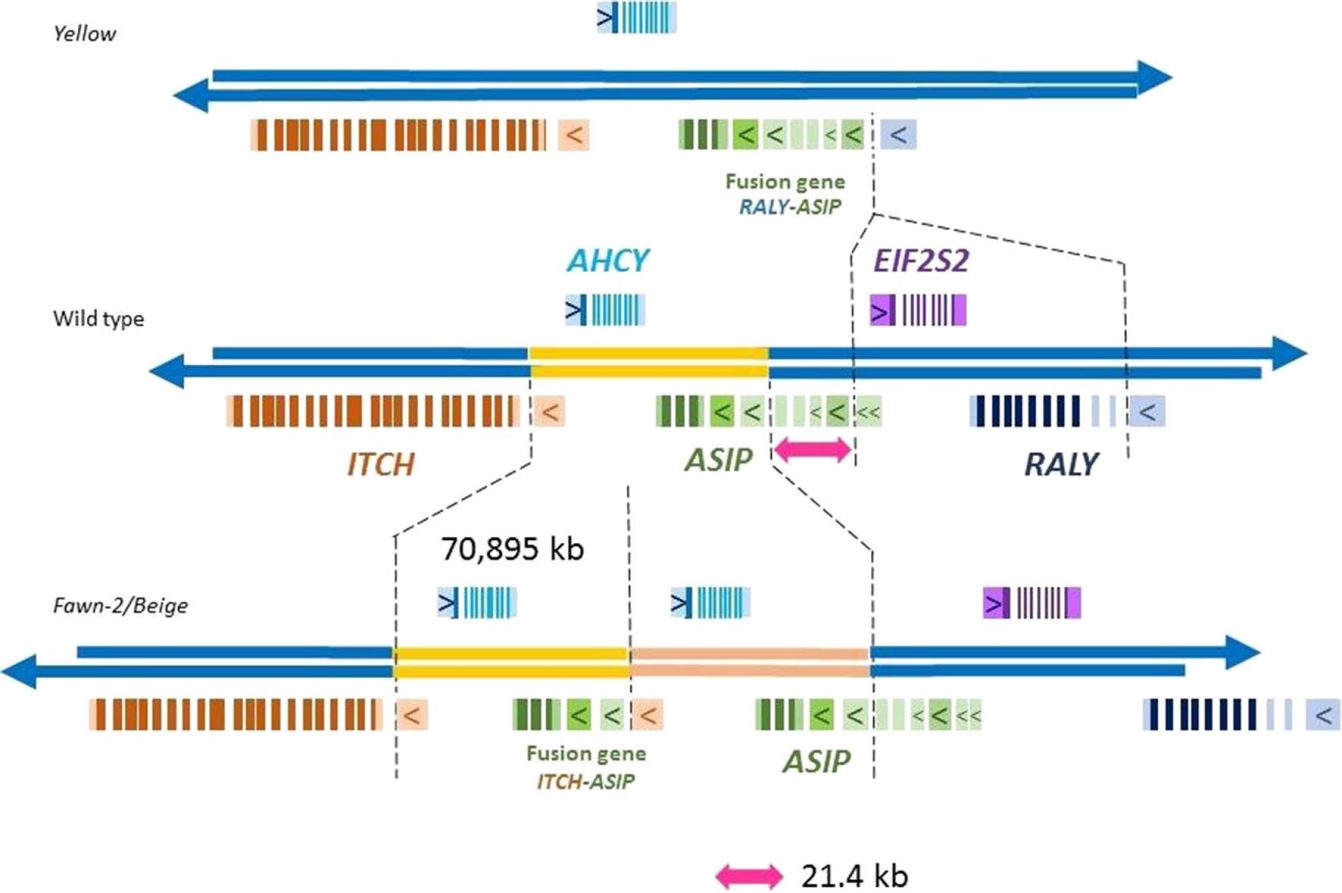
*ASIP* locus in yellow and fawn-2/beige quail genomes. The deletion characterized in yellow and the tandem duplication identified in fawn-2/beige quails concern the same region. The gene organization on the reference genome is drawn in the middle. The two structural events both lead to the formation of a fusion gene. As on Fig. 1, 5′ UTR exons that could be the starting site of a transcript are symbolized by a rectangle with an arrow. In animals  heterozygous for the *yellow* allele, the fusion gene *RALY*-*ASIP* can produce a fusion transcript [15] coding for a normal ASIP protein. In animals carrying the *fawn*-2/*beige* allele, the fusion gene *ITCH*-*ASIP* can produce three possible transcripts coding for the ASIP protein from the three exons including a TSS

The breakpoints of this 70,895-bp tandem duplication (NC-029535 [1,371,395(or 98)–1,442,295 (or 98)]) are located downstream of the *ITCH* exon 1 and upstream of the *ASIP* exon e4 (5′ UTR) (Fig. 3). The 70,895 bp of the duplicated region include the entire *AHCY* gene on one strand, and parts of the *ASIP* and *ITCH* genes on the other strand. Thus, in this rearrangement, complete copies of the original genes remain. Indeed, animals that carry this duplication have one normal copy and an extra portion of the *ASIP* gene [two 5′ UTR exons (e4 and e5) and three coding exons], which are inserted just downstream of the *ITCH* non-coding exon 1 (5′UTR) (Fig. 3), thus forming an *ITCH*-*ASIP* fusion gene. This fusion gene has several possible TSS: *ITCH* exon-1, *ASIP*-TSS-2 and *ASIP*-TSS-3 (Figs. 1, 3).

### Transcription of the region carrying the *fawn-2/beige* mutation

In 2008, Hiragaki et al. [4] analyzed the expression of *ASIP* in the skin of neonatal chicks, but in the current study, we sampled embryonic skin to study the phenotypic consequences of the *fawn*-*2/beige* mutation at an earlier stage, since they are already visible at hatch. All possible transcripts from this region were sequenced from 15-day embryo skin samples. The fusion gene has three possible TSS, i.e. in the first exon (5′UTR) of *ITCH* and the TSS-containing *ASIP* exons e4 (TSS2) and e5 (TSS3) (Figs. 1, 3). Transcripts of the fusion gene from the TSS-containing *ASIP* exons e4 and e5 (ASIP-tr2 and ASIP-tr3) could not be distinguished from those of the normal *ASIP* copy because there was no sequence difference. RT-PCR with primers that are located respectively in the 5′UTR of *ITCH* and in the coding exons of *ASIP* and sequencing of the products showed that the fusion transcript includes *ITCH* exon 1 and the three *ASIP* coding exons but not the *ASIP* exons e4 and e5. Since the 5′UTR exon 1 of *ITCH* does not contain a start codon, no fusion protein is expected from this fusion transcript, which supports our hypothesis that the protein produced from the *ITCH*-*ASIP* fusion gene is a normal ASIP protein.

We also performed quantitative PCR to evaluate the expression level of each ASIP transcript and the impact of the fusion gene on the amount of transcripts coding for the ASIP protein. We designed primers to selectively amplify three types of ASIP transcripts: the fusion transcript ITCH-ASIP, which can be transcribed only from the *ITCH*-*ASIP* fusion gene, and ASIP-tr2 and ASIP-tr3, which can be transcribed from both the *ASIP* gene and the *ITCH*-*ASIP* fusion gene. In addition, we used a primer pair to evaluate the overall amount of all ASIP-coding transcripts (see Additional file 3 and Additional file 4: Table S1).

**Fig. 4:**
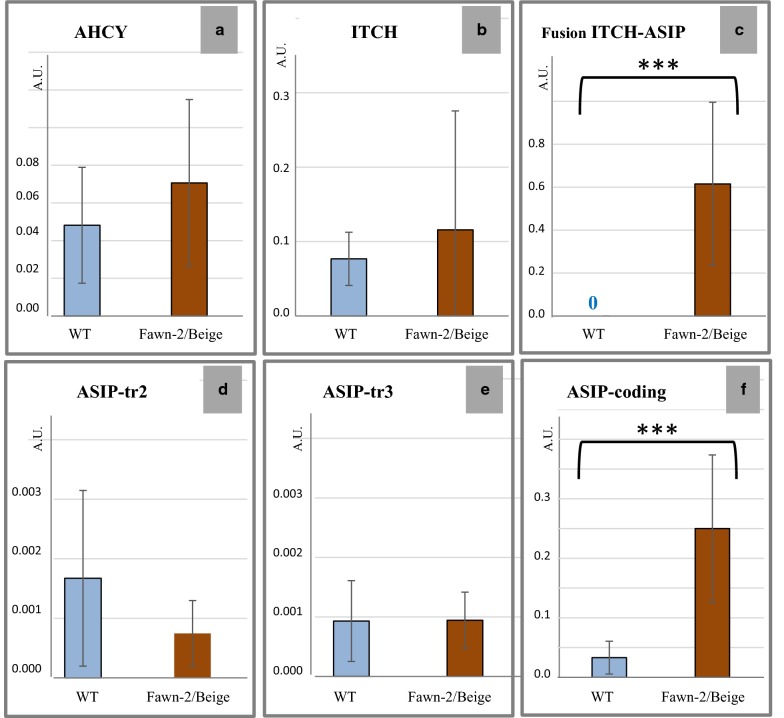
Quantitative expression of the transcripts from the *ASIP* region in fawn-2/beige versus wild-type quails 15-days embryos (skin). Blue: wild-type embryos [n = 10, excepted for ASIP-tr2 (n = 6) and ASIP-tr3 (n = 8)]. Brown: embryos homozygous for the *fawn*-2/*beige* mutation (n = 9, except for ASIP-tr2 and ASIP-tr3 where n = 7). The abundance of transcripts is expressed in arbitrary units (AU), values are mean ± standard deviation. ***Significant difference with *p* value < 0.001. The different graphs represent the transcription from: **a** *ITCH*; **b** *AHCY*; **c** the *ITCH*-*ASIP* fused gene; **d** ASIP-tr2 and **e** ASIP-tr3 representing alternative transcripts from the *ASIP* and *ITCH*-*ASIP* fused genes; **f** overall amount of all ASIP-coding transcripts

The differences in amounts of AHCY or ITCH transcripts were not significant between wild-type and fawn-2/beige animals (Fig. 4a, b). Although the fawn-2/beige animals carried two copies of the *AHCY* gene because of the duplication, there was no difference in the expression level of *AHCY* in the skin of embryos.

Similar to Hiragaki et al. [4], we observed a statistically significant difference in ASIP-coding transcripts between wild type quails and fawn-2/beige quails (Fig. 4f) and, as expected, the ITCH-ASIP fusion transcript was detected only in the fawn-2/beige quails (Fig. 4c). It should be noted that qPCR does not allow the comparison of the expression of two genes *A* and *B*, which means that we were not able to determine if there was a difference in expression level between the fusion transcript ITCH-ASIP and the ASIP or ITCH transcripts. Nevertheless, we compared their expression between individuals (see Table 2) and found no significant difference in the amounts of ASIP-tr2 (Fig. 4d) or ASIP-tr3 transcripts (Fig. 4e) between wild-type and fawn-2/beige animals, but ASIP-coding transcripts were significantly more abundant in fawn-2/beige than in wild-type animals (Fig. 4f), which can be attributed to the ITCH-ASIP fusion transcript. Our findings agree with those reported by Hiragaki et al. [4] (see Table 2) and show that the difference in the amount of ASIP-coding transcripts between fawn-2/beige and wild-type animals was much larger than expected with a simple gene duplication. They also indicate that this high expression of the ITCH-ASIP fusion transcript could explain the large excess in coding transcripts for the ASIP protein in skin samples.

**Table 2:** Relative expression of ASIP-coding transcripts

Study	Sample	Wild-type	Fawn-2/beige
		(Mean ± SD)	(Mean ± SD)
Hiragaki et al. [4]	Neonatal chicks	1.00 ± 0.35	12.99 ± 9.49
	Dorsal skin	3 animals (triplicate)	3 animals (triplicate) homozygous for *fawn*-*2*
Current study	15-days embryos	1.00 ± 0.83	7.55 ± 3.73
	Dorsal skin	10 animals (duplicate)	9 animals (duplicate) homozygous for *beige*

## Discussion

The 141,162-bp deletion that we identified for the *yellow* mutation confirms previous results [15]. It leads to the creation of a *RALY*-*ASIP* fusion gene and deletion of the *EIF2S2* gene. The strong similarity between the *lethal yellow* mutation in mouse [5–7] and the *yellow* mutation in quail, as previously suspected by Nadeau et al. [15], is confirmed at the genomic level. However, our study reveals that the deletion is also combined with a 241-bp insertion, which is mainly composed of repeated sequences that do not originate from this region. Nadeau et al. (2008) showed that this structural modification preserves the coding structure of the *ASIP* gene but affects the regulatory 5′ region, and results in the production of transcripts from the *RALY*-*ASIP* fusion gene [15]. However the expression of the transcripts coding for ASIP is disorganized with no over-expression in the skin [4, 15]. The transcription of the fusion gene depends on the promoter of the *RALY* gene but its 5′ region may also contain alternative regulatory sequences. In addition, we cannot exclude the possibility that the loss of the 5′ region of the *ASIP* gene has additional impacts.

Concerning the *fawn*-2/*beige* mutation, a first conclusion is that *fawn*-2 and *beige* share the same allele at the *ASIP* locus, and precedence should be given to the name *fawn*-2 for these variations. No conclusion can be drawn for the other *fawn* allele since no molecular data is available for this phenotype. We identified a 71,895-bp duplication (Fig. 3) and although this leads to a full duplication of *AHCY,* its expression remains unchanged. In contrast, an increased transcription of *ASIP* is described as responsible for the fawn-2/beige phenotype [4]. We show that the fawn-2/beige animals studied here carry two copies of *ASIP*, one normal and one truncated (Fig. 3) just upstream of the TSS-2 (*ASIP*-TSS-2), which generates a fusion gene between the 5′UTR exon 1 of *ITCH* and the three coding exons of *ASIP* (Fig. 3). Thus, there is an increased production of ASIP-coding transcripts in the skin, which partly explains the lighter colour of their feathers. However, we also show that the increase in the amount of ASIP transcripts is higher than would be expected from a simple duplication of *ASIP* (Table 2), and we suggest that the 5′ region of the fusion gene has a higher transcription rate than that of *ASIP* alone. We found that the *ITCH* promoter is active for the fusion gene, but we were not able to compare the efficiency of the transcription of *ITCH* and the transcription of the fusion gene. Nevertheless, we cannot exclude the possibility that the loss of the 5′ region of the *ASIP* gene itself, putatively containing repressor sequences, could have additional impacts.

The *ASIP* region is particularly well conserved in birds and in mammals, both in terms of gene order and orientation. In Merino sheep, Norris and Whan [11] showed that a duplication with breakpoints located downstream from the *ITCH*-exon-1 and upstream from the *ASIP* coding exons and resulting in an *ITCH*-*ASIP* fusion transcript causes the dominant white|tan phenotype. This rearrangement is very similar to that of the allele we identified in the fawn-2/beige quails, except that, in the dominant white|tan sheep phenotype, the *ASIP* gene from the original segment appears to be inactivated. We found no evidence of such an effect in the fawn-2/beige quails. In addition, Norris and Whan [11] detected a SINE-type repeat element at the 5′ and 3′ breakpoints of their duplication and found that the genome of white|tan sheep contained several repetitions of this segment. For the fawn-2/beige quail, we identified only a micro-homology (Fig. 2b) between 5′BKPT-*fawn*-*2* and 3′BKPT-*fawn*-*2*. Such events occur very often in duplicated regions and result from the mechanism involved in the duplication process [26].

The similarities between the *yellow* mutations in mice and quails and between the *fawn*-2/*beige* mutation in quails and the dominant *white*|*tan* mutation in sheep, raise the question of whether two independent deletion/duplication events in the same region can occur by chance or whether the presence of some conserved genomic features make this region prone to deletion/duplication events. The early/late feathering phenotype, which is due to a large duplication (*K* mutation) in chicken [27, 28], and to a 5-bp frameshift deletion in turkey [29], is another example of such events. Several other mutations involving the *ASIP* gene have been described in other species. In the white Alpaca, a promoter from another gene (*NCOA6*) was identified upstream of the *ASIP* coding exons, which could originate from an inverted duplication such as that characterized in agouti mice [12], without any other duplication or deletion [13]. In Normande cattle that carry the *A*^*br*^ allele, a full-length L1-B1 element inserted in the 5′ UTR sequence of *ASIP* was identified as responsible for the over-expression of *ASIP* [8]. The *viable yellow* mutation in mouse is due to the insertion of an intracisternal A particle (IAP) type retrotransposon into the promoter region of the *ASIP* [9]. Although numerous structural genomic modifications in the 5′ UTR region of *ASIP* are known, we list only those that are not neutral. All of these (*agouti*/mouse, *A*^*br*^/Normande cattle, *white*/alpaca, *white*|*tan*/sheep, *viable yellow*/mouse) are dominant gain-of-function mutations, which support the hypothesis that the 5′ region of *ASIP* could contain negative regulatory sequences that are active in the skin. This is an additional argument in favour of the loss of repressor sequences in the genome fawn-2/beige quails, which could have an impact on the expression of the fusion gene in addition to the effects brought by the 5′ region of *ITCH* itself. The loss of a 141-kb region in yellow quails (NC-029535 [1,463,709–1,604,872]) leads to a much diluted feather colour phenotype compared to fawn-2/beige quails. The 5′ region of the *ITCH*-*ASIP* fusion gene could also result from a 71-kb deletion (NC-029535 [1,371,395 (or 98)–1,442,295 (or 98)]). Our study suggests that the 21.4-kb region in quail (NC-029535 [1,442,295–1,463,709], indicated by a double pink arrow on Figs. 1b, 3), which contains the *ASIP* 5′UTR e1, e2, Ne9 and e3 exons (Fig. 1b), could include these repressor sequences. We hope that, in the near future, the improved annotation of livestock genomes resulting from the efforts of the FAANG consortium [30], will allow us to examine the hypothesis of repressor sequences in the 21.4-kb region.

## Conclusions

We have refined the characterization of a 141-kb long deletion in heterozygous quails for the *yellow* allele and identified a 71-kb long tandem duplication in the *ASIP* region in fawn-2/beige quails. We conclude that *fawn*-2 and *beige* are the same allele, and due to precedence, these should now both be given the name *fawn*-2. We have shown that the loss (*yellow*) or the pseudo-loss (*fawn*-2/*beige*) of two distinct regions upstream of the coding exons of *ASIP* leads to the formation of a fusion gene that result in the production of the ASIP transcript, and especially for *fawn*-*2* allele, at a higher rate. Although the fusion gene benefits from a new promoter and a new 5′ region that may contain alternative up-regulatory sequences, we cannot exclude the possibility that the loss of the 5′ region of *ASIP* itself has additional impacts. Indeed, the hypothesis of the loss of repressor sequences within *ASIP* should also be considered, since several structural modifications localized upstream of the *ASIP* coding sequences have been reported in mouse, alpaca, cattle, sheep and now quail, which all cause dominant gain-of-function phenotypes.

## Additional files

**Additional file 1.** Phenotypes of Japanese quail: wild-type and homozygous *beige/beige* females, homozygous *beige/beige* males and a heterozygous *yellow*/*WT* female.

**Additional file 2.** Alignment of sequence reads [21, 25].

**Additional file 3.** Additional details for the study of transcripts [31, 32].

**Additional file 4.** List of primers and sequences used in this study.

**Additional file 5.** Examination of read alignments for *yellow*, *fawn*-2 and *beige* sequenced genomes.
