# Supplementary material for: Two new structural mutations in the 5′ region of the ASIP gene cause diluted feather color phenotypes in Japanese quail
Source: Genet Sel Evol. 2019 Apr 15;51:12. doi: 10.1186/s12711-019-0458-6 (PMC6466734; doi:10.1186/s12711-019-0458-6)
Supplement: Supplementary file 1 — Additional file 1. Phenotypes of Japanese quail: wild-type and homozygous beige/beige females, homozygous beige/beige males and a heterozygous yellow/WT female. [file 12711_2019_458_MOESM1_ESM.docx]

**Additional file 1 Figure S1**

Phenotypes of Japanese quail: wild-type and homozygous *beige/beige* females, homozygous *beige/beige* males and a heterozygous *yellow*/*WT* female

**Females *WT* and *beige/beige***


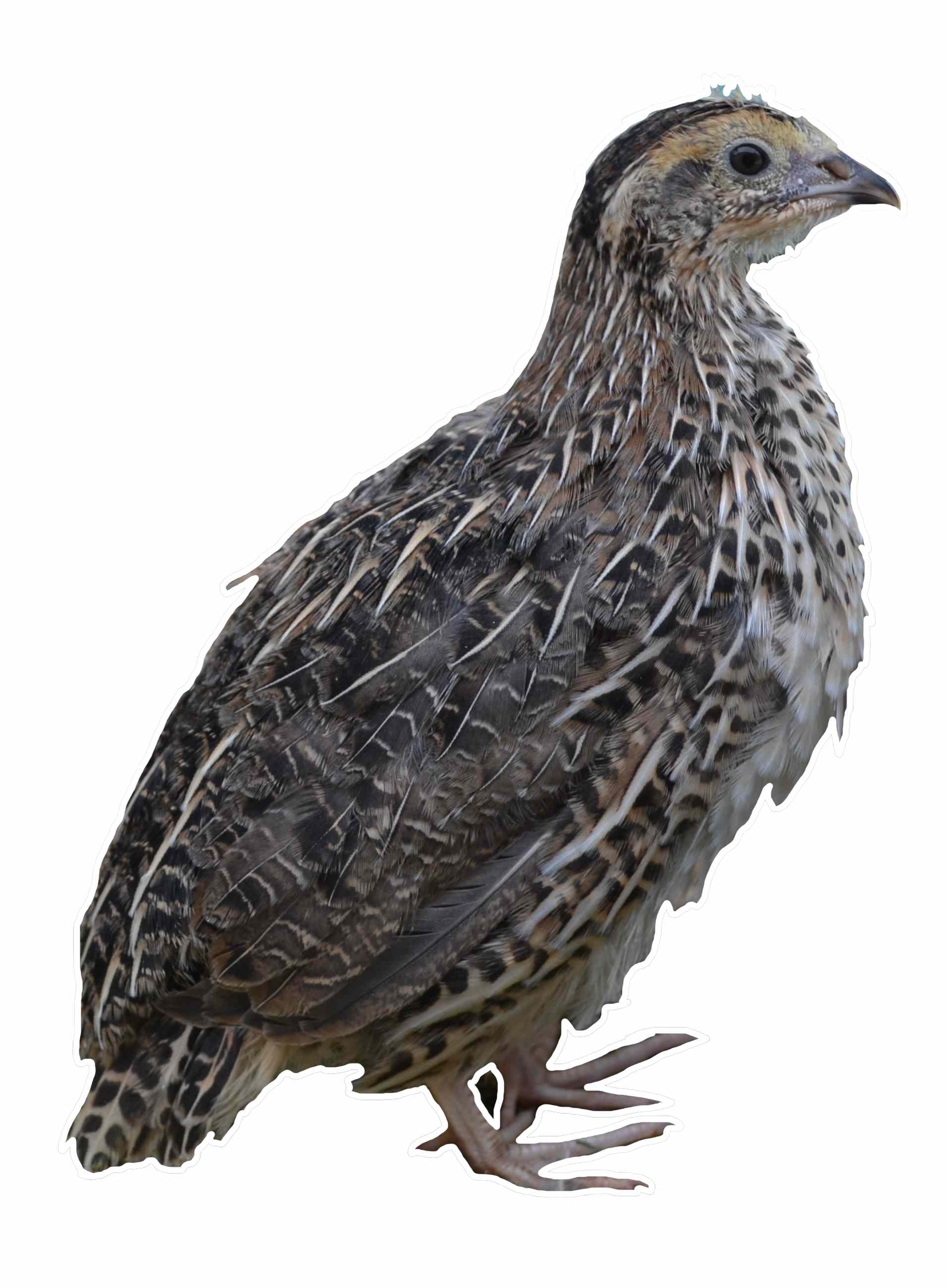

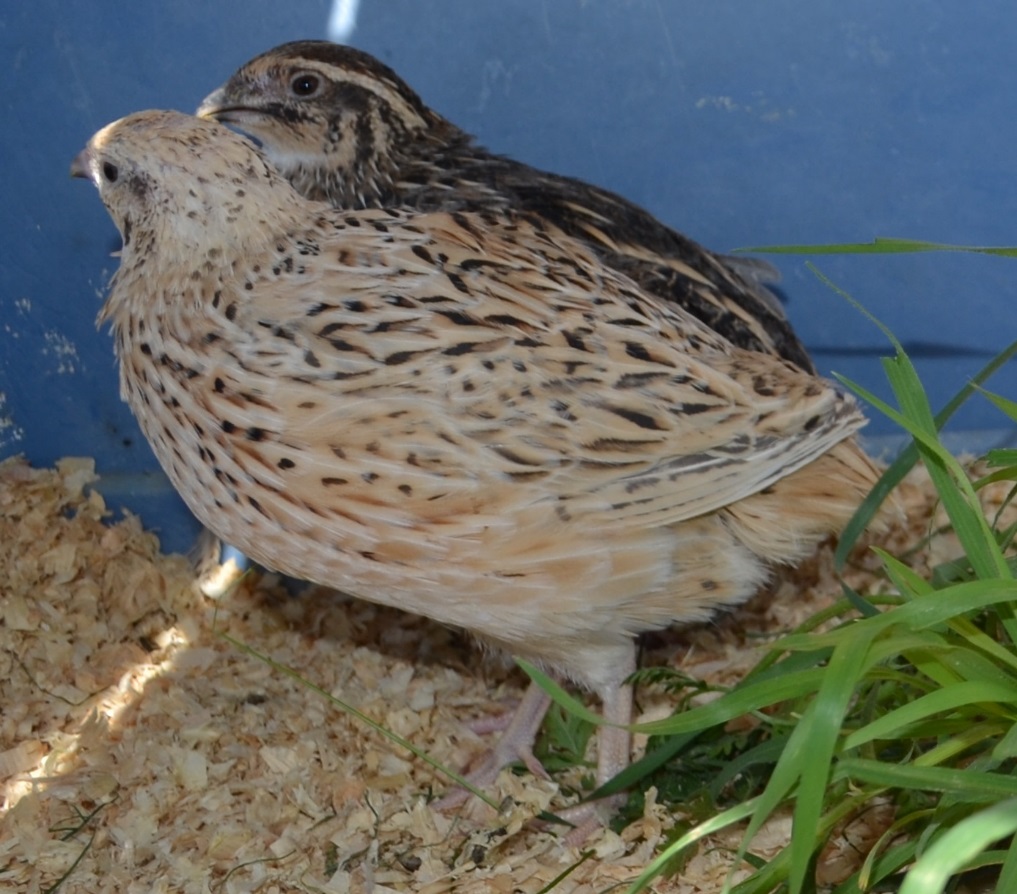


WT female One beige/beige and one WT female

**Homozygous *beige/beige* (male)**


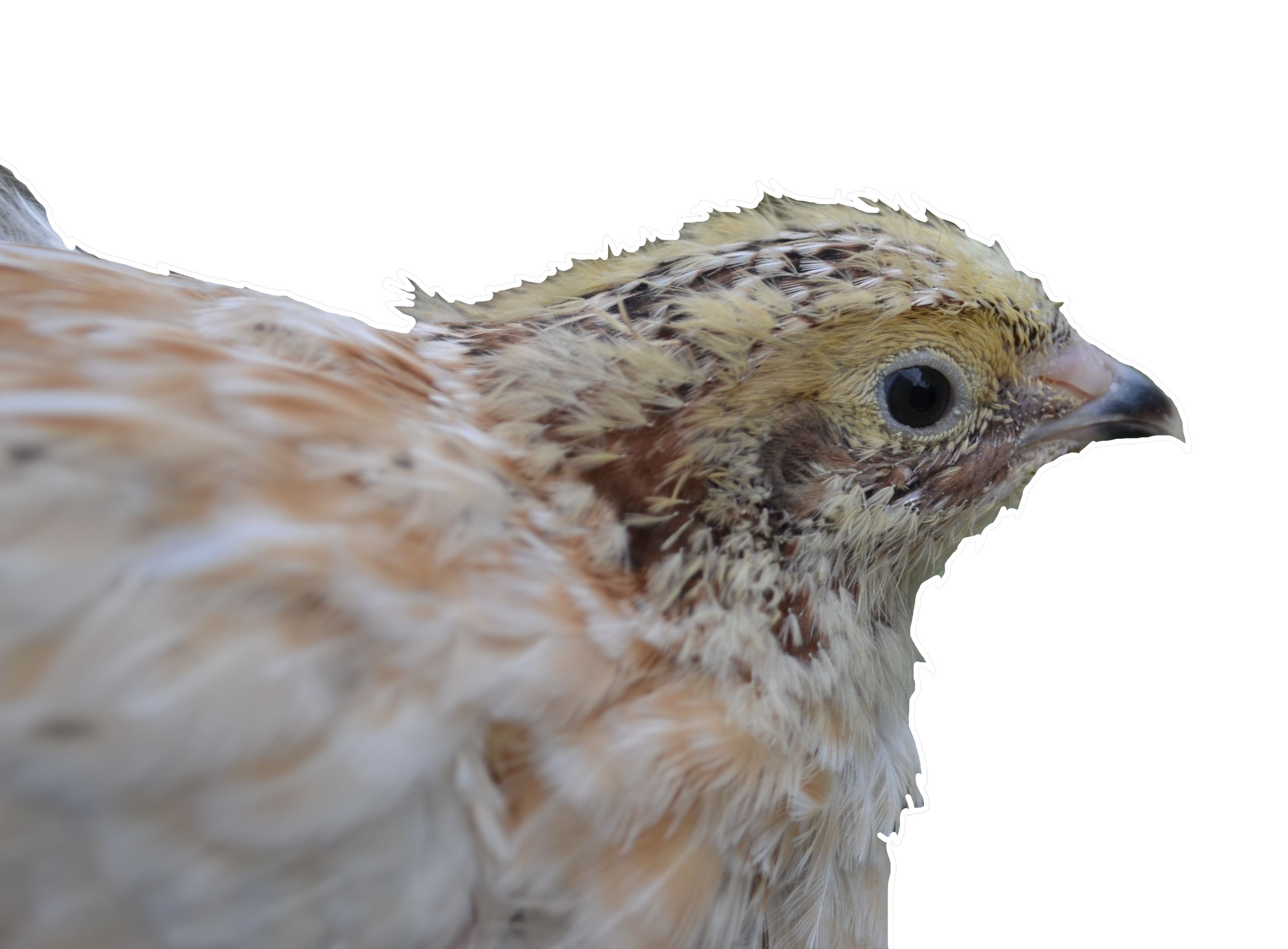

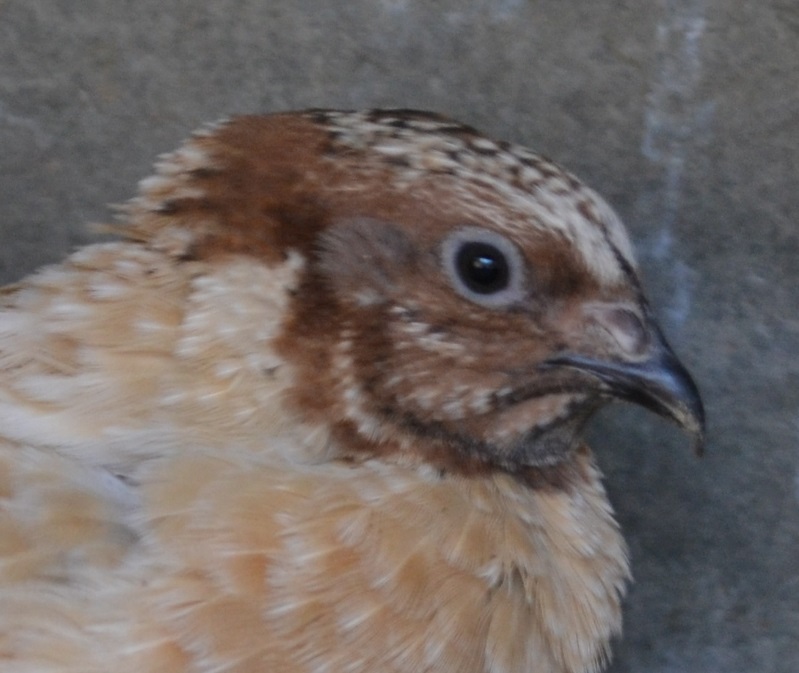


Homozygous beige/beige male at 4 weeks Homozygous beige/beige male at 8 months


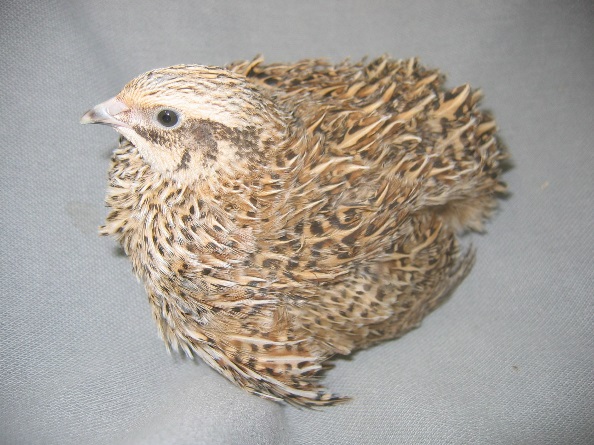


**Heterozygous *yellow*/*WT* female**
