# Supplementary material for: Two new structural mutations in the 5′ region of the ASIP gene cause diluted feather color phenotypes in Japanese quail
Source: Genet Sel Evol. 2019 Apr 15;51:12. doi: 10.1186/s12711-019-0458-6 (PMC6466734; doi:10.1186/s12711-019-0458-6)
Supplement: Supplementary file 2 — Additional file 2. Alignment of sequence reads [21, 25]. [file 12711_2019_458_MOESM2_ESM.docx]

**Additional file 2**

**Alignment of sequence reads**

The sequence data is deposited on SRA, under Accession SRR8224502 and SRR8224503 for the two heterozygous yellow animals and SRR8224504 for the fawn-2 and SRR8224505 for the Beige individuals. Bioproject PRJNA506074.

We used the BWA-MEM program to align the 150 bp reads to the reference genome [2[1](#_ENREF_1)]. To visualize the reads aligned against the reference genome sequence, we used the Integrative Genome Viewer program, IGV <http://software.broadinstitute.org/software/igv/>. Classically, the pairs of reads are aligned head to head to the considered locus of the reference genome with a fragment size compatible with the size selection carried out before the sequencing. In addition this software offers an evaluation of the amount of sequences aligned at each position (read depth). When a region including a deletion is sequenced, we may observe paired-ends both mates mapped at two distanced localizations on the same chromosome and a decrease of depth in the deleted region. When a region including tandem duplication is sequenced we can observe paired-ends (P.E.) aligned tail to tail to the considered locus of the reference genome and an increase of depth in the duplicated region. BWA-MEM can propose the mapping of some reads as split reads. These split reads identified on IGV were aligned individually by BLAST (Basic Local Alignment Search Tool) to the quail genome (<https://blast.ncbi.nlm.nih.gov/Blast.cgi>) [[25](#_ENREF_2)].
