# Supplementary material for: Two new structural mutations in the 5′ region of the ASIP gene cause diluted feather color phenotypes in Japanese quail
Source: Genet Sel Evol. 2019 Apr 15;51:12. doi: 10.1186/s12711-019-0458-6 (PMC6466734; doi:10.1186/s12711-019-0458-6)
Supplement: Supplementary file 3 — Additional file 3. Additional details for the study of transcripts [31, 32]. [file 12711_2019_458_MOESM3_ESM.docx]

**Additional file 3**

**Additional details for the study of transcripts**

**RNA and reverse transcription**

Twenty-five beige and 25 wild-type plumage quail eggs were incubated in an electric hatchery under appropriate conditions. Embryos were harvested after 15 days of incubation, whereas the normal incubation time in quail is between 16 and 19 days. Skin samples from the right pectoral muscle were frozen immediately in liquid nitrogen and stored at -80°C, for RNA and DNA extractions. A PCR test on DNA was used for sex determination as described in [[22](#_ENREF_1)] and five samples of each sex were selected for each of the two phenotypes for further RNA analyses.

Samples for RNA preparation were disrupted, homogenized and ground into a fine powder by rapid agitation for 1 min, in a liquid-nitrogen-cooled grinder with stainless steel beads before RNA extraction. Total RNAs were extracted with TRIzol reagent (Invitrogen) [[31](#_ENREF_2)], purified using the NucleoSpin (Machery-Nagel) RNA kit, and treated with DNase to remove contaminating DNA. Reverse-transcription was performed with the high-capacity cDNA reverse transcription kit (Applied Biosystems #4368814) using 2 μg of total RNA from each sample as a template and dN_6_ random primers, according to the manufacturer’s instructions.

**RT-PCR and qRT-PCR**

PCR primers were designed using Primer3 (<http://bioinfo.ut.ee/primer3-0.4.0/> ).

Primers for analysing the various DNA breakpoints and junctions are in Table S1 [see Additional file 3 Table S1]. PCR were performed under standard procedures. Primers for qPCR were designed from sequences of two different exons for all genes to avoid amplification of genomic DNA. The tested genes were *ACHY*, *ITCH*, *ASIP* and the two reference genes *RPS13* and *GAPDH*. In addition, primers for testing an ITCH-ASIP fusion transcript were designed [see Table S1]. Quantitative PCR were performed on the ABI 7900HT (Sequence Detection System 7900HT) with Sybr-green in a 384-well plate. All measurements were performed in duplicate on the same plate. The transcript concentrations were corrected with respect to the reference genes (we considered the geometric mean between *RPS13* and *GAPDH* results). Data were analysed with the ABI software to obtain Ct values (threshold cycle). Six points of dilutions of a mix of cDNA were used for each gene, to determine PCR efficiency. Since efficiency levels were similar for all measured genes (including the reference genes), results were expressed as 2^(Ct_ref – Ct_gene)^ x1000 in arbitrary units.

It is important to underline that each PCR point was performed with only two primers, even if ITCH and ITCH-ASIP have a common upper primer and ASIP-tr2, ASIP-tr3, and ITCH-ASIP have a common lower primer.

All primers used are described in Additional file 3 Table S1.

Sequencing of PCR fragments was performed (ABI3700) as already described [32].
