# Supplementary material for: Two new structural mutations in the 5′ region of the ASIP gene cause diluted feather color phenotypes in Japanese quail
Source: Genet Sel Evol. 2019 Apr 15;51:12. doi: 10.1186/s12711-019-0458-6 (PMC6466734; doi:10.1186/s12711-019-0458-6)
Supplement: Supplementary file 4 — Additional file 4. List of primers and sequences used in this study. [file 12711_2019_458_MOESM4_ESM.docx]

**Additional file 4 Table S1**

**List of primers and sequences used in this study**

| **Amplicon** | **Primers sequences and features** | |
| --- | --- | --- |
| JUNCT-*Yel* | GGATGATGGAAAGGGGATTC | GCACTGGTCTCGGGTGTAAA |
| 3'BKPT-*Yel* | TGTGAAACCACCGAAGATCC | GCACTGGTCTCGGGTGTAAA |
| 5'BKPT-*Yel* | GGATGATGGAAAGGGGATTC | TCACAACCAAAGCCTCCTGT |
| 5'BKPT-*fawn-2* | GAGGATGTTAGGGATGGCATT | CTCATTGCAGTCTCTGGGAAG |
| 3'BKPT-*fawn-2* | TGCTTGGATGGAAACAATCAC | CAGGAATTATAAAAGCTCCAGGAA |
| JUNCT-*fawn-2* | CAGGAATTATAAAAGCTCCAGGAA | ACCACAGATTGCAGCCTTTT |
| AHCY | GGCTGAATCAAAATGCGGTA | *AHCY*-ex4 (coding exon) |
| qPCR | TGACCAGTCTGCCTTCTGCT | *AHCY*-ex5 (coding exon) |
| ITCH-ASIP | AGGGACGGAGGAGCTGAAC | *ITCH*-ex1 (5’ UTR exon) |
| qPCR | TCCCACTGTCATGAAGACCA | *ASIP*-Ce1 (first coding exon) |
| ITCH | AGGGACGGAGGAGCTGAAC | *ITCH*-ex1 (5’ UTR exon) |
| qPCR | CCCACCAGGTACTCCTGACTT | *ITCH*-ex2 (first coding exon) |
| ASIP-tr2 | TCACCAGCATTTGCATGTTT | *ASIP*-e4 (5’ UTR exon) |
| qPCR | TCCCACTGTCATGAAGACCA | *ASIP*-Ce1 (first coding exon) |
| ASIP-tr3 | TTTCGCATCCTACACCATTTG | *ASIP*-e5 (5’UTR exon) |
| qPCR | TCCCACTGTCATGAAGACCA | *ASIP*-Ce1 (first coding exon) |
| ASIP-coding | ATCTCCCACCCATCTCCATC | *ASIP*-Ce1 (first coding exon) |
| qPCR | TGGGGGTGTCTTCAGTTCAG | *ASIP*-Ce3 (third coding exon) |
| GAPDH | GGAGAAACCAGCCAAATATGATG |  |
| qPCR reference transcript | AGGTGGAAGAATGGCTGTCA |  |
| RPS13 | AAGAAGGCTGTTGCTGTTCG |  |
| qPCR reference transcript | CTTCCAGTTGGGTGGCAGTA |  |
