# Supplementary material for: Two new structural mutations in the 5′ region of the ASIP gene cause diluted feather color phenotypes in Japanese quail
Source: Genet Sel Evol. 2019 Apr 15;51:12. doi: 10.1186/s12711-019-0458-6 (PMC6466734; doi:10.1186/s12711-019-0458-6)
Supplement: Supplementary file 5 — Additional file 5. Examination of read alignments for yellow, fawn-2 and beige sequenced genomes. [file 12711_2019_458_MOESM5_ESM.docx]

**Additional file 5**

**Examination of reads alignments for Yellow, *fawn-*2 and *beige* sequenced genomes**

**Figure S2**

**Examination of read alignments for the *yellow* sequenced genome**

Using IGV software, each read pair is symbolized by two large arrows separated by a thin line. When a region including a deletion is sequenced, we may observe paired-ends (P.E.) with both mates mapped at two distanced localizations on the same chromosome. The IGV view (in the purple box) shown here represents alignments of reads from a heterozygous Yellow quail, with each read of the two paired ends. In brick color, the two P.E. aligned in two regions separated by 141 kb. When the alignment of a read is segmented (split read) we expect to find a segment on either side of the deletion. The split reads identified on IGV were aligned individually by *BLAST* on the quail genome.


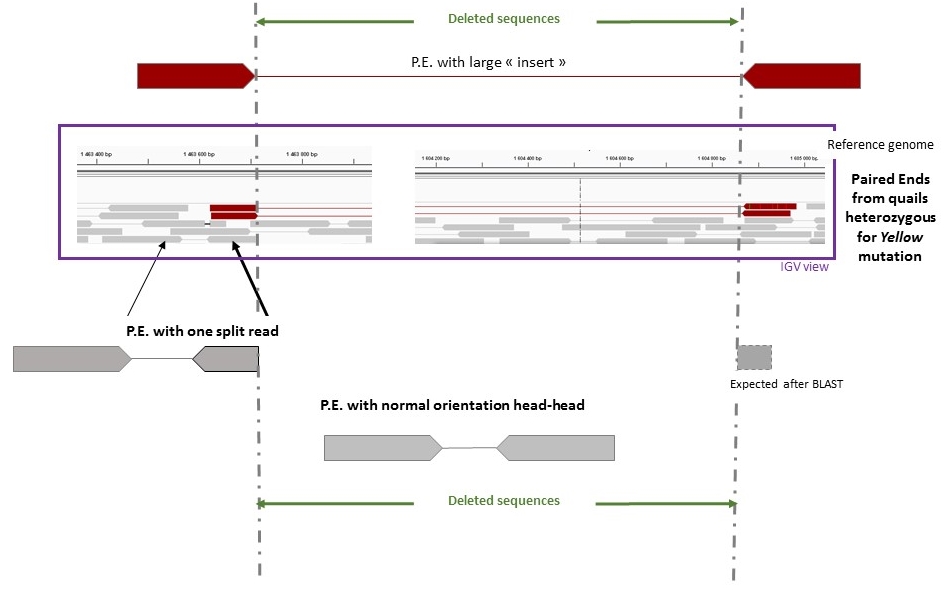


**Figure S3**

**Examination of reads alignments for *fawn-*2 and *beige* sequenced genomes**

The IGV view shows alignments of reads from homozygous fawn-2 and beige quails where the tail-tail oriented reads are separated by a fragment of ~71 kb. Each read pair is symbolized by two arrows separated by a thin line (brick color). Only tandem duplications are likely to generate read pairs that are positioned in the opposite direction (tail-tail) and separated by large inserts (brick color). The large number of reads in this region and the visualization of paired ends with opposite orientation suggest the existence of a 71-kb tandem duplication.


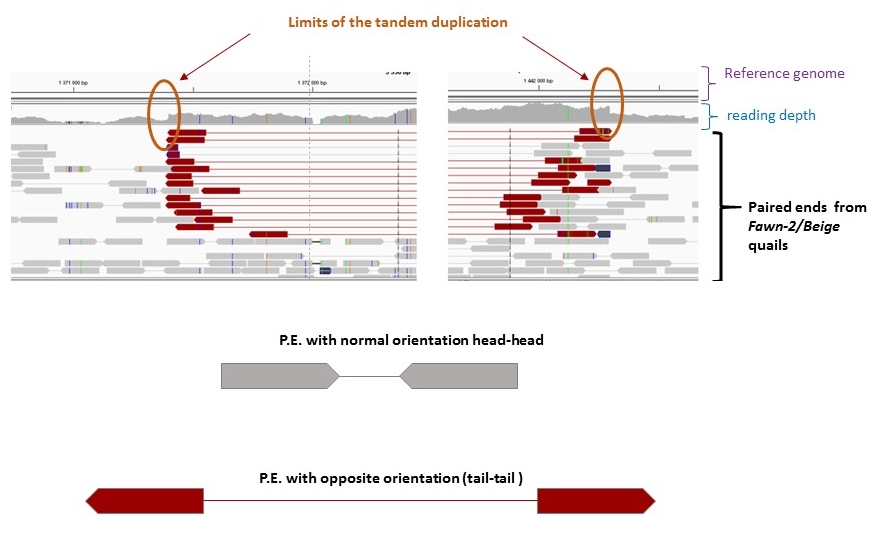


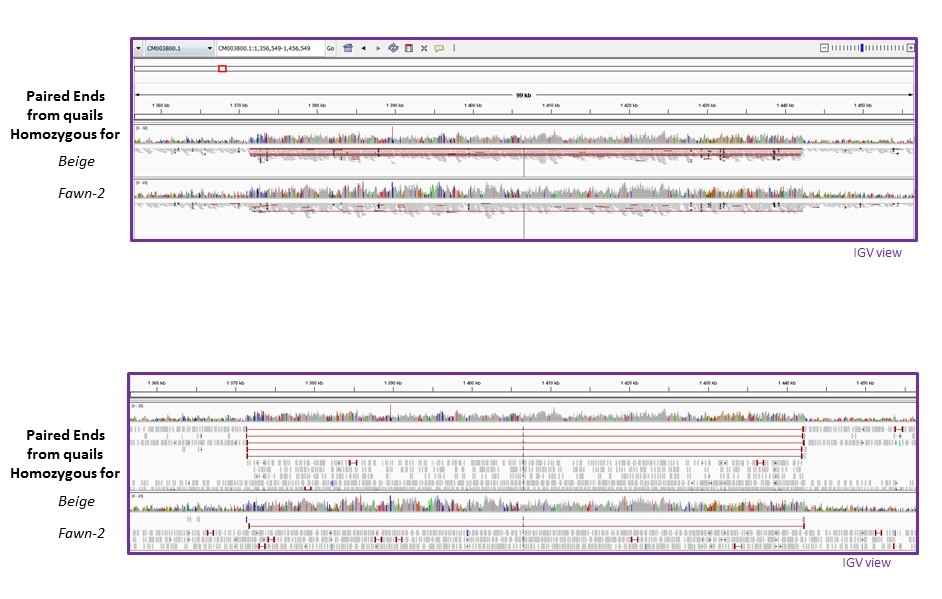


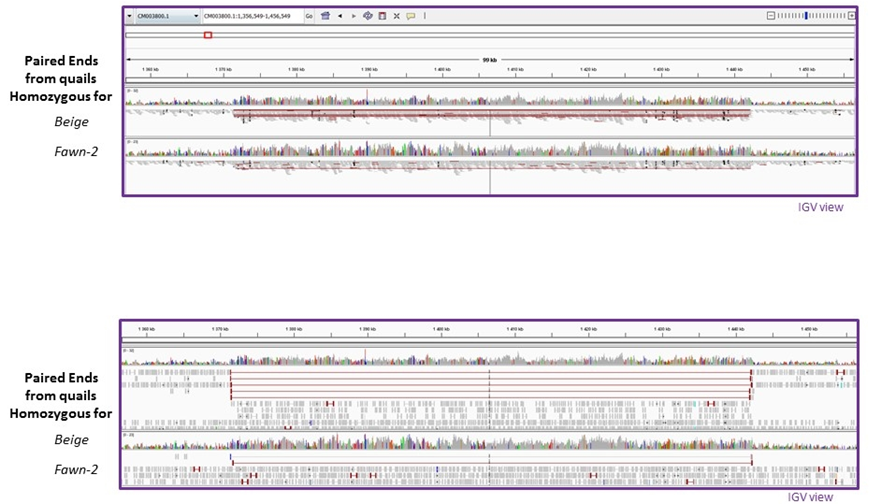


The difference of reading depth is in favor of a simple tandem duplication thus the possibility of three or four copies of this region was excluded.
